# Ocular symptoms in COVID-19 infection: a survey study

**DOI:** 10.1186/s12348-022-00319-w

**Published:** 2022-12-20

**Authors:** Matthew McHarg, Yujuan Wang, Mehmet Yakin, Alex Zeleny, Sonny Caplash, H Nida Sen, Shilpa Kodati

**Affiliations:** 1 National Eye Institute, National Institutes of Health, 10 Centre Drive, 10/10N248, Bethesda, MD USA; 2 George Washington University School of Medicine and Health Sciences, Washington, DC USA; 3 Department of Ophthalmology, University of Health Sciences, Ankara Training and Research Hospital, Ankara, Turkey; 4 Georgetown University School of Medicine, Washington, DC USA; 5 Department of Ophthalmology, University of Pittsburgh Medical Center, Pittsburgh, PA USA

**Keywords:** COVID-19, Survey study, Ocular surface, Infection, Inflammation

## Abstract

**Background:**

Coronavirus disease 2019 (COVID-19) systemic symptoms and sequelae have been studied extensively, but less is known about the characterization, duration, and long-term sequelae of ocular symptoms associated with COVID-19 infection. The purpose of this study was to analyze the frequency, spectrum, and duration of ocular symptoms in participants with COVID-19 infection treated in inpatient and outpatient settings.

**Methods:**

A retrospective electronic survey was distributed to NIH employees and the public who reported testing positive for SARS-CoV-2. The anonymous survey collected information on demographics, past ocular history, systemic COVID-19 symptoms, and ocular symptoms.

**Results:**

A total of 229 (21.9% male and 78.1% female, mean age 42.5 ± 13.9) survey responses were included. Ocular symptoms were reported by 165 participants with a mean of 2.31 ± 2.42 symptoms. The most commonly reported ocular symptoms were light sensitivity (31.0%), itchy eyes (24.9%), tearing (24.9%), eye redness (24.5%), and eye pain (24.5%). Participants with ocular symptoms had a higher number of systemic symptoms compared to participants without ocular symptoms (mean 9.17 ± 4.19 vs 6.22 ± 3.63; OR: 1.21; 95% CI: 1.11 – 1.32; *p* < 0.001). Ocular symptoms were more common in those who reported a past ocular history compared to those who did not (81.8% vs 67.1%; OR: 2.17; 95% CI: 1.08 – 4.37; *p* = 0.03). Additionally, the onset of ocular symptoms occurred most frequently at the same time as systemic symptoms (47.5%), and 21.8% reported symptoms lasting ≥ 14 days.

**Conclusions:**

Ocular surface-related symptoms are the most frequent ocular manifestations, and systemic disease severity is associated with the presence of ocular symptoms. Additionally, our results show that ocular symptoms can persist post-COVID-19 infection. Further work is needed to better understand ocular symptoms in COVID-19 and long-term sequelae.

## Background

A number of ocular manifestations have been reported in association with the novel severe acute respiratory syndrome coronavirus 2 (SARS-CoV-2), including conjunctivitis, chemosis, and uveitis, amongst others [1–4]. Although reported rates of ocular symptoms have varied, a recent meta-analysis by Inomata et al. reported that 11.2% of coronavirus disease 2019 (COVID-19) patients experienced some form of ocular manifestation [5]. Another meta-analysis of COVID-19 patients by Loffredo and colleagues reported the frequency of conjunctivitis, the most commonly described ocular manifestation of SARS-CoV-2 infection, as 5.9% [6, 7].

Currently, several theories exist regarding the pathogenesis of ocular symptoms associated with COVID-19 infection. Notably, the ocular surface has been implicated as a potential route of entry of SARS-CoV-2 virus through the nasolacrimal system [8, 9]. Additionally, conjunctival expression of angiotensin-converting enzyme 2 (ACE2), which mediates viral entry into the host cell, and transmembrane serine protease 2 (TMPRSS2), which cleaves the virus’s spike (S) protein, may facilitate an ocular route of viral entry [10]. Although SARS-CoV-2 has also been detected via reverse transcription polymerase chain reaction (RT-PCR) on conjunctival swabs, evidence of spread via ocular secretions is still inconclusive [11, 12].

Despite these reports, more information is still needed on the frequency, timing, spectrum, and duration of SARS-CoV-2-associated ocular symptoms. Additionally, the majority of published reports describing ocular symptoms involve hospitalized patients, whereas comparatively less is known about those treated in the outpatient setting. Thus, the purpose of this study is to systematically characterize the range of ocular symptoms found in majority non-hospitalized individuals with COVID-19 infection.

## Methods

A survey was developed using the online program Research Electronic Data Capture (REDCap), hosted within the National Institute of Diabetes and Digestive and Kidney Diseases (NIDDK). REDCap is a secure, web-based application designed to support data capture for research studies; it was developed and is licensed by Vanderbilt University [13]. Responses were collected and managed using REDCap’s survey tools, and consulting support was provided by the Biomedical Translational Research Information System (BTRIS). Both NIDDK and BTRIS are part of the National Institutes of Health (NIH).

Our survey was designed to gather a large amount of information regarding ocular symptoms in COVID-19 infection. The study information was distributed to NIH employees who had tested positive for SARS-CoV-19, as well as the general public through social media and patient recruitment mailing lists. The survey queried participants about demographics, past ocular and medical history, details of their COVID-19 infection, and ocular symptoms. All responses were kept anonymous, and survey responses were collected from October 2020 through June 2021. In order to participate in the study, respondents were required to consent to completing the survey, confirm testing positive for SARS-CoV-2 via a polymerase chain reaction (PCR) test, and attest that they were 18 years of age or older. This study was approved as exempt by the NIH Office of IRB Operations, and patients or the public were not involved in the design, or conduct, or reporting, or dissemination plans of this project.

Only complete responses were included for analysis. For statistical analyses, SPSS v.17.0 statistical software for Windows (SPSS Inc., Chicago, IL) was used. For descriptive statistics, continuous variables are presented as means ± standard deviations, and categorical variables are presented as frequencies and proportions. Multivariable logistic regression models were used to identify significant predictors of clinical symptoms adjusted by age, gender and race. Comparisons of continuous variables between two variables were done by student t-test for normally distributed variables. For variables without normal distribution, Mann–Whitney U test was used for comparisons between two independent variables. A two-tailed *p* value of < 0.05 was considered significant.

## Results

Following exclusion of ineligible participants and incomplete responses, 229 (21.9% male and 78.1% female, mean age 42.5 ± 13.9 years) complete survey responses were included for analysis from both hospitalized (10.0%) and non-hospitalized (90.0%) respondents (Table 1).

**Table 1:** Participant demographics. Significant differences (*p* < 0.05) between hospitalized and non-hospitalized participant demographics are displayed in bold text

**Demographics**	Hospitalized	Non-hospitalized	Total
	(*n* = 23)	(*n* = 206)	(*n* = 229)
**Age (mean ± SD)**	**49.1 ± 15.8**	**41.8 ± 13.6**	42.5 ± 13.9
**Gender**			
**Female**	15	163	178 (78.1%)
**Male**	8	42	50 (21.9%)
**Unknown**	0	1	1 (0.6%)
**Race/Ethnicity**			
**White non-Hispanic**	16	142	158 (69.0%)
**Black**	1	24	25 (10.9%)
**Asian**	4	18	22 (9.6%)
**White Hispanic**	0	13	13 (5.7%)
**Other**	2	9	11 (4.8%)
**Country of residence**			
**US**	20	196	216 (94.3%)
**Other**	3	10	13 (5.7%)
**Employment**			
**Healthcare worker**	**4 (17.4%)**	**82 (39.8%)**	86 (37.6%)
**NIH employee**	**1 (4.3%)**	**73 (35.4%)**	74 (32.3%)

NIH employees and health-care workers accounted for 32.3% and 37.6% of responses, respectively, and 30.1% were tested as part of a healthcare employee testing program. Most participants resided in the United States (94.3%). A total of 77 patients (33.6%) reported a past ocular history (Table 2), including most frequently conjunctivitis (11.4%) and dry eyes (10.9%). Further, 27 participants (11.8%) reported using eyedrops including artificial tears, lubricating ointments, and/or anti-allergy drops before the onset of their symptoms.

**Table 2:** Past ocular history reported by survey participants

**Past Ocular History**	Total (*n* = 229)
Conjunctivitis	26 (11.4%)
Dry eye	25 (10.9%)
Other	19 (8.3%)
Cataract	12(5.2%)
Allergic Eye Disease	7 (3.1%)
Strabismus	6 (2.6%)
Eye Injury	3 (1.3%)
Double vision	2 (0.9%)
Retinal detachment	2 (0.9%)
Macular degeneration	2 (0.9%)
Uveitis	2 (0.9%)
Sudden irreversible vision loss	1 (0.4%)
Thyroid eye disease	1 (0.4%)
Blindness	0
Color Blindness	0
Glaucoma	0
Sudden reversible vision loss	0
Diabetic retinopathy	0

All hospitalized participants (mean number of systemic symptoms: 10.00 ± 4.45) and 98.5% of non-hospitalized participants (mean number: 8.16 ± 4.19) experienced at least one COVID-19 associated systemic symptom. When separated by gender, the number of reported systemic symptoms was significantly higher in females than males (8.69 ± 4.39 vs 7.20 ± 3.49, respectively; *p* = 0.03).

Ocular symptoms were reported by 165 (72.1%) participants (mean number of ocular symptoms per participant: 2.31 ± 2.42; range 0–11). The most frequently reported ocular symptoms were light sensitivity (*n* = 71, 31.0% of all respondents), itchy eyes (*n* = 57, 24.9%), tearing (*n* = 57, 24.9%), eye redness (*n* = 56, 24.5%), eye pain (*n* = 56, 24.5%), blurred vision (*n* = 52, 22.7%), and foreign body sensation (*n* = 43, 18.8%). Ocular symptoms were predominantly bilateral (90.9%). A summary of ocular symptom data can be found in Table 3.

**Table 3:** Ocular symptoms reported by survey participants. Significant differences (*p* < 0.05) between hospitalized and non-hospitalized ocular symptoms are displayed in bold text

**Ocular symptoms**	Hospitalized	Non-hospitalized	Total
	(*n* = 23)	(*n* = 206)	(*n* = 229)
**Light sensitivity**	**12 (52.2%)**	**59 (28.6%)**	71 (31.0%)
**Tearing**	**10 (43.5%)**	**47 (22.8%)**	57 (24.9%)
**Itchy eyes**	6 (26.1%)	51 (24.8%)	57 (24.9%)
**Redness**	**10 (43.5%)**	**46 (22.3%)**	56 (24.5%)
**Pain**	6 (26.1%)	50 (24.3%)	56 (24.5%)
**Blurred vision**	**10 (43.5%)**	**42 (20.4%)**	52 (22.7%)
**Foreign body sensation**	5 (21.7%)	38 (18.4%)	43 (18.8%)
**Mucous discharge**	**8 (34.8%)**	**33 (16.0%)**	41 (17.9%)
**Floaters**	**7 (30.4%)**	**29 (14.1%)**	36 (15.7%)
**Flashes of light**	**6 (26.1%)**	**17 (8.3%)**	23 (10.0%)
**Eyelid swelling**	3 (13.0%)	15 (7.3%)	18 (7.9%)
**Blind spots**	**3 (13.0%)**	**7 (3.4%)**	10 (4.4%)
**Double vision**	**4 (17.4%)**	**6 (2.9%)**	10 (4.4%)

When separated by inpatient and outpatient settings, ocular symptoms were reported in 20 (87.0%) hospitalized respondents compared to 145 (70.4%) non-hospitalized respondents (OR: 3.50; 95% CI: 0.94 – 13.02; *p* = 0.06). The mean number of ocular symptoms per respondent was also significantly greater in hospitalized participants than in their non-hospitalized counterparts (3.91 ± 3.09 vs 2.14 ± 2.28, respectively; *p* = 0.01). Lastly, ocular symptoms were more common in those who reported a past ocular history compared to those who did not (81.8% vs 67.1%; OR: 2.17; 95% CI: 1.08 – 4.37; *p* = 0.03).

The association between ocular and systemic symptoms was examined in-depth (Tables 4 and 5). Systemic disease severity was associated with the presence of ocular symptoms, as participants with ocular symptoms had a higher number of systemic symptoms compared to participants without ocular symptoms (mean 9.17 ± 4.19 compared to 6.22 ± 3.63; OR: 1.21; 95% CI: 1.11 – 1.32; *p* < 0.001). Additionally, the onset of ocular symptoms occurred most frequently at the same time as systemic symptoms (47.5%) when compared to before (17.5%) and after systemic symptoms (35.0%).

**Table 4:** Relationship between systemic symptoms and any ocular symptoms adjusted for age, gender, and race. Significant results (*p* < 0.05) are displayed in bold text

Systemic Symptom	Symptom present?	Any ocular symptom	OR*	95% CI*	*P*-value*
**Measured fever > 100.4 F**	**No**	94 (66.2%)	1		
	**Yes**	71 (81.6%)	**2.31**	**1.18 – 4.52**	**0.02**
**Subjective fever**	**No**	95 (69.9%)	1		
	**Yes**	70 (75.3%)	1.32	0.71 – 2.43	0.38
**Chills**	**No**	65 (62.5%)	1		
	**Yes**	100 (80.0%)	**2.27**	**1.23 – 4.19**	**0.01**
**Muscle aches**	**No**	41 (57.7%)	1		
	**Yes**	124 (78.5%)	**2.46**	**1.32 – 4.59**	**0.01**
**Joint pain**	**No**	86 (64.7%)	1		
	**Yes**	79 (82.3%)	**2.28**	**1.17 – 4.42**	**0.02**
**Runny nose**	**No**	78 (65.0%)	1		
	**Yes**	87 (79.8%)	**2.14**	**1.14 – 4.01**	**0.02**
**Sore throat**	**No**	88 (68.2%)	1		
	**Yes**	77 (77.0%)	1.65	0.89 – 3.06	0.12
**Cough**	**No**	49 (62.0%)	1		
	**Yes**	116 (77.3%)	**1.94**	**1.05 – 3.60**	**0.03**
**Shortness of breath**	**No**	99 (69.2%)	1		
	**Yes**	66 (76.7%)	1.47	0.77 – 2.79	0.24
**Wheezing**	**No**	131 (68.6%)	1		
	**Yes**	34 (89.5%)	**4.25**	**1.39 – 13.01**	**0.01**
**Chest pain**	**No**	112 (65.5%)	1		
	**Yes**	53 (91.4%)	**5.81**	**2.13 – 15.84**	**0.001**
**Fatigue/Tiredness**	**No**	29 (56.9%)	1		
	**Yes**	136 (76.4%)	**2.48**	**1.23 – 4.97**	**0.01**
**Nausea/Vomiting**	**No**	113 (68.5%)	1		
	**Yes**	52 (81.3%)	1.71	0.82 – 3.56	0.15
**Headache**	**No**	46 (63.9%)	1		
	**Yes**	119 (75.8%)	1.80	0.95 – 3.40	0.07
**Abdominal pain**	**No**	135 (68.2%)	1		
	**Yes**	30 (96.8%)	**13.16**	**1.73 – 99.92**	**0.01**
**Diarrhea**	**No**	103 (67.3%)	1		
	**Yes**	62 (81.6%)	**2.26**	**1.13 – 4.55**	**0.02**
**Loss of taste**	**No**	67 (67.0%)	1		
	**Yes**	98 (76.0%)	1.50	0.82 – 2.73	0.19
**Loss of smell**	**No**	62 (66.7%)	1		
	**Yes**	103 (75.7%)	1.55	0.85 – 2.83	0.15
**Other**	**No**	129 (68.3%)	1		
	**Yes**	36 (90.0%)	**4.07**	**1.34 – 12.38**	**0.01**
*Adjusted for age, gender and race

**Table 5:** Relationship between any ocular symptoms and age, gender, and race

	**OR**	**95% CI**	***P****-value*
Age	1.01	0.99—1.03	0.37
Gender			
Male	1		
Female	1.78	0.90—3.51	0.10
Race / ethnicity			
White non-Hispanic	1		
White Hispanic	0.62	0.19—2.02	0.43
Black	1.89	0.61—5.88	0.27
Asian	0.50	0.20—1.28	0.15
Others	1.12	0.28—4.50	0.87

There was no significant difference in the number of respondents who reported ≥ 1 ocular symptom when the responses were divided into age groups (18–29 years, 30–49 years, ≥ 50 years). When specific eye symptoms were analyzed, however, foreign body sensation was reported significantly more frequently in the ≥ 50 age group (OR: 4.81; 95% CI: 1.47 – 15.74; *p* = 0.01), and blurred vision was significantly common in 30 – 49 and ≥ 50 age groups (OR: 4.85; 95% CI: 1.46 – 16.08; *p* = 0.01 and OR: 7.99; 95% CI: 2.30 – 27.80; *p* = 0.001 respectively).

Respondents were also asked about whether their ocular symptoms were ongoing or had resolved at the time of their responses. Of the participants who reported resolved symptoms, the mean duration of symptoms was 16.2 days ± 50.6. A total of 31 (18.8%) respondents reported ongoing ocular symptoms with a mean duration of 73.6 days (± 104.6). Amongst all respondents who endorsed ocular symptoms, both resolved and ongoing, 36 (21.8%) had eye symptoms lasting ≥ 14 days (range 14–400 days; mean 2.28 ± 1.86 symptoms per participant). Of these, 3 participants were hospitalized and 33 were non-hospitalized. The most frequently reported ocular symptoms lasting ≥ 14 days were blurred vision (*n* = 14, 8.4% of respondents with ocular symptoms), floaters (*n* = 9, 5.5%), eye redness (*n* = 8, 5.5%), tearing (*n* = 8, 5.5%), and sensitivity to light (*n* = 8, 5.5%). All 13 ocular symptoms were reported at least once, and each ocular symptom had at least one respondent who experienced persistent symptoms for ≥ 14 days.

Notably, only 22 (13.3%) of respondents with ocular symptoms sought medical attention by an eye care professional (ophthalmologist or optometrist). Of those participants who sought care, 9 out of 22 (40.9%) respondents had their eye symptoms attributed to COVID-19 infection by their eye care professional.

## Discussion

Our results demonstrate several points of interest, including 1) ocular surface related symptoms are the most frequently reported ocular symptoms in COVID-19 infection; 2) ocular symptoms are more frequent in those with a reported past ocular history; 3) systemic disease severity is associated with the presence of ocular symptoms; and 4) a proportion of participants reported post-COVID-19 persistent ocular symptoms.

In large-scale studies, the reported prevalence of ocular findings in COVID-19 infection have varied from as low as 1.4% to as high as 11.2% [5, 14, 15]. However, Inomata and colleagues suggested that these numbers are likely an underestimation, as individuals with COVID-19 have a range of manifestations and may be unlikely to seek out ophthalmic evaluation when other life-threatening symptoms are present. The most frequently observed signs in a meta-analysis by La Distia Nora et al. were epiphora, conjunctival injection, and chemosis, which are commonly seen in other forms of viral conjunctivitis [16]. Other reported ocular findings have included both anterior and posterior segment findings such conjunctivitis, uveitis, acute macular neuroretinopathy, and retinopathy [17, 18]. Notably, ocular manifestations have occasionally been described as the sole or initial presentation of SARS-CoV-2 infection [19–21]. The majority of large series studies that investigate ocular manifestations of COVID-19 are in hospitalized patients, and differentiating these manifestations from factors related to hospitalization rather than the infection itself, especially with intensive care, can be challenging. Thus, our study provides insight into ocular symptoms experienced by COVID patients who were—in large part—not hospitalized (90.0%).

Although survey and cross-sectional studies have reported varying frequencies of ocular symptoms, current literature points towards a predominance of ocular surface symptoms when eye symptoms are present. A recent meta-analysis by Soltani et al. found that the most prevalent ocular symptoms were dry eyes (23.8%) and eye pain (10.3%) [22]. Similarly, Nasiri et al., in their meta-analysis, found the most common ocular manifestations to be dry eyes/foreign body sensation (16.0%), redness (13.3%), tearing (12.8%), itching (12.6%), and eye pain (9.8%) [23]. Although varied, these analyses are largely consistent with the ocular symptoms most commonly reported in our study, including light sensitivity (31.0%), itchy eyes (24.9%), tearing (24.9%), and eye redness/pain (24.5%).

Corneal and conjunctival expression of ACE2 receptor, a known entry mechanism of the SARS-CoV-2, has been postulated as a potential mechanism for direct viral invasion of the ocular surface and subsequent ocular manifestations [10, 24]. Additionally, Zhong and colleagues revealed a pooled positivity rate of 3.9% from conjunctival swabs [16]; however, there are numerous studies that have reported patients with positive conjunctival swabs but no ocular symptoms, and vice versa [1, 12, 25]. Further, a review by Douglas and colleagues concluded there is no clear relationship between conjunctival titers and transmissibility [2]. Overall, current research indicates that the relationship between PCR positivity in conjunctival swabs, transmission, and ocular symptoms remains unclear.

Reports have also found that patients with severe disease were more likely to have associated ocular manifestations, presumably due to a higher viral load leading to disseminated disease [16, 26]. Our study yielded similar results, showing that participants with ocular manifestations had 9.17 ± 4.19 systemic symptoms, while those without ocular symptoms had 6.22 ± 3.63 systemic symptoms (*p* < 0.001).

The majority of participants in our study (47.5%) reported the onset of ocular symptoms at the same time as systemic symptoms. However, 17.5% of reported ocular symptoms presented prior to the onset of systemic symptoms. These results are consistent with La Distia Nora and colleagues’ study and the meta-analysis by Inomata et al., who reported prodromal ocular symptoms in 28% and 12.5% of cases, respectively [5, 15]. These findings highlight the need for ophthalmologists to maintain a high degree of suspicion when evaluating patients with ocular surface complaints during the COVID-19 pandemic.

There is a paucity of data in the literature regarding ocular symptoms associated with “long-haul” COVID-19 infection. A significant proportion of participants with ocular symptoms (21.8%) in our study reported eye symptoms lasting ≥ 14 days, including most frequently blurred vision, floaters, eye redness, tearing, and sensitivity to light. Pardhan and colleagues reported eye symptoms lasting ≥ 14 days in 20% of their cohort [27], and Vallejo-Garcia et al. observed persistent conjunctivitis in 9.4% of their patients with a mean follow up time of 6 weeks after the initial positive COVID-19 test [28]. Notably, in the study by Vallejo-Garcia and colleagues, conjunctival swabs were negative in all patients with persistent ocular symptoms, which may suggest that the ocular symptoms are not the result of active infection. Other than these few reports, there is little data regarding post-COVID-19 infection ocular symptoms, and potential mechanisms are still being investigated. Indeed, it remains unclear whether the mechanisms of systemic “long-haul” COVID-19 infection, which have been attributed viral persistence in ACE2-expressing organ systems, autoimmunity due to cryptic antigens and viral mimicry, and persistent inflammation due to the altered cytokine environment and persistence of pro-inflammatory immune cells, can explain the persistence of ocular symptoms [29].

This study has several limitations: As with any survey study, there is unavoidable recall bias and there are varying levels of symptom awareness between survey participants. There is also likely an inclusion bias towards respondents experiencing eye symptoms, as this survey was distributed by the National Eye Institute. This bias at least partially accounts for the high frequency of ocular symptoms in our study (72.1%). Another potential limitation is that our study reported ocular *symptoms* of COVID-19 experienced by patients, not *diagnoses* since the respondents were not examined by an ophthalmologist at the time of survey completion. Lastly, the results of this study would have been strengthened by a larger and more representative sample size.

In conclusion, our results show that ocular surface-related symptoms were more common and vision-affecting symptoms were rare. The majority of participants reported the onset of ocular symptoms at the same time as systemic symptoms, and over a fifth of our respondents reported ocular symptoms lasting ≥ 14 days in duration. As vaccination rates increase, hospitalization rates fall, and outpatient cases rise; we believe these results are critical to the understanding of COVID-19 and its ocular manifestations, particularly in outpatient settings. Overall, further research is needed to fully comprehend the pathophysiology and sequalae of ocular symptoms associated with COVID-19 infection.

## Data Availability

The datasets used and/or analysed during the current study are available from the corresponding author on reasonable request.

## Abbreviations

SARS-CoV-2: Severe acute respiratory syndrome coronavirus 2
COVID-19: Coronavirus disease 2019
ACE2: Angiotensin-converting enzyme
TMPRSS2: Transmembrane serine protease 2
RT-PCR: Reverse transcription polymerase chain reaction
REDCAP: Research Electronic Data Capture
NIDDK: National Institute of Diabetes and Digestive and Kidney Diseases
BTRIS: Biomedical Translational Research Information System
NIH: National Institutes of Health
